# SeaTrack-DeepSORT: A Vision-Based Ship Detection and Multi-Object Tracking Framework for Complex Maritime Environments

**DOI:** 10.3390/s26196072

**Published:** 2026-09-25

**Authors:** Junjie Gao, Liang Hong, Sibo Zhang

**Affiliations:** School of Energy and Power Engineering, Nanjing University of Science and Technology, Nanjing 210094, China

**Keywords:** ship detection, multi-object tracking, YOLOv11, DeepSORT, unmanned surface vehicle, maritime perception

## Abstract

With the rapid development of intelligent maritime systems and unmanned surface vehicles (USVs), accurate ship detection and multi-object tracking in complex maritime environments have become essential for autonomous perception and intelligent navigation. However, challenges such as scale variations, background interference, illumination changes, target occlusions, and camera motion significantly degrade tracking performance. To address these issues, this paper proposes SeaTrack-DeepSORT, a maritime detection and tracking framework that connects multiscale detection with confidence-dependent state updates and motion-compensated association. Its SeaDet-YOLO detector incorporates ADown downsampling and an additional P2 detection head into YOLOv11. The DeepSORT-based tracker combines quadratic measurement-noise scaling, ECC-Affine correction of predicted target centers, and two-stage association, using detection confidence to guide both association and the weighting of matched observations. Extensive experiments on multiple maritime datasets show that SeaTrack-DeepSORT improves detection and tracking performance while maintaining stable and continuous trajectories. The resulting framework supports autonomous perception and intelligent monitoring for unmanned surface vehicles.

## 1. Introduction

With the increase in maritime traffic density and the growing demand for intelligent maritime monitoring, vision-based ship detection and tracking have become important research directions in ocean engineering, port security, waterway supervision, and autonomous maritime systems [1,2]. Particularly for unmanned maritime platforms such as unmanned surface vehicles (USVs), reliable visual perception capability serves as an essential foundation for environmental understanding, target monitoring, and autonomous navigation [3]. Compared with traditional sensing approaches, such as radar and the Automatic Identification System (AIS), visual sensors can acquire richer information on target appearance, spatial location, and motion states, while offering advantages including lower equipment costs, higher information dimensions, and stronger environmental adaptability [4]. Therefore, video-based ship detection and tracking technologies have significant application value in coastal monitoring, port inspection, maritime safety supervision, and intelligent maritime perception [5].

In recent years, with the development of deep learning and computer vision technologies, target detection methods based on deep neural networks have gradually become important approaches for maritime visual perception. Compared with traditional methods based on handcrafted features, deep learning-based detection algorithms can automatically learn high-level semantic features of targets and exhibit stronger adaptability under complex backgrounds and scale variations. Redmon et al. [6] proposed the YOLO series algorithms, which achieve object detection through an end-to-end regression approach and achieve a favorable balance between detection accuracy and real-time performance, providing an important technical foundation for real-time maritime target detection. Subsequently, lightweight detection models such as YOLOv3, YOLOv7, and YOLOv8 further optimized network architectures and improved real-time detection capabilities, making them more suitable for resource-constrained unmanned platforms [7,8].

To address the unique challenges in maritime environments, including a high proportion of small targets, significant target scale variations, and complex background interference, researchers have further conducted studies on network optimization for ship detection. For example, Yang et al. [9] applied the YOLO method to ship detection in remote sensing images and verified the effectiveness of deep learning-based approaches for maritime target recognition. Prasad et al. [10] constructed a dataset for maritime visual tasks, providing an important foundation for water surface target detection and recognition research. In recent years, researchers have further improved detection performance in complex maritime environments through lightweight network architectures, multi-scale feature fusion, and attention mechanisms. Yang et al. [11] proposed the TAD-YOLO method, which addresses the issues of large ship scale variations and limited computational resources in unmanned surface vehicle (USV) maritime monitoring scenarios. By designing a lightweight network architecture, the proposed method improves detection efficiency and achieves favorable detection accuracy and real-time deployment capability on multiple maritime ship datasets. Ruan et al. [12] proposed Marine-YOLO, an improved maritime target detection algorithm based on YOLOv11. By introducing multi-scale feature enhancement modules and attention mechanisms, the proposed method improves small object detection capability in complex maritime environments while maintaining low computational complexity and enhancing detection accuracy. Wang et al. [13] proposed a method that integrates multi-scale attention features and an improved feature fusion structure to enhance the feature representation of ship targets in complex maritime environments, thereby improving the detection accuracy of multi-scale ship targets. Although the aforementioned methods have achieved promising results in maritime ship detection tasks, object detection algorithms mainly focus on target localization and classification in individual frames and cannot provide target identity information and motion trajectories in continuous video sequences. For applications such as maritime monitoring, autonomous perception of USVs, and intelligent navigation, detection results alone are insufficient to satisfy the requirements for continuous target tracking and behavior analysis. Therefore, combining object detection algorithms with multi-object tracking (MOT) algorithms has become an important approach for achieving continuous perception of maritime targets.

In the field of multi-object tracking (MOT), the Tracking-by-Detection framework has become the mainstream technical approach. Among them, Bewley et al. [14] proposed the SORT algorithm, which utilizes a Kalman filter to predict the motion states of targets and employs the Hungarian algorithm for target association, achieving efficient online multi-object tracking. Subsequently, Wojke et al. [15] proposed the DeepSORT method, which introduces deep appearance features to enhance target identity association capability and effectively reduces identity switches under conditions of target occlusion and crossing motion. However, DeepSORT mainly relies on fixed motion models and appearance matching, making it still vulnerable to variations in target motion, detection errors, and camera motion in complex dynamic scenarios. To overcome the limitations of traditional MOT algorithms in complex environments, researchers have proposed a series of improved approaches in recent years. For example, Zhang et al. [16] proposed ByteTrack, which improves target trajectory recovery under occlusion conditions by utilizing low-confidence detection results through a two-stage association strategy. Aharon et al. [17] proposed BoT-SORT, which integrates camera motion compensation (CMC), appearance features, and motion information to improve tracking performance in complex scenarios. Cao et al. [18] proposed OC-SORT, which improves tracking robustness under occlusion and nonlinear motion by incorporating observation-centric motion modeling and association strategies.

However, most existing multi-object tracking algorithms are mainly designed for terrestrial traffic or pedestrian scenarios and do not explicitly consider the coupled effects encountered in maritime videos. For an on-board camera, platform motion is superimposed on the motion of the vessels, causing global image displacement and reducing the reliability of trajectory prediction. Meanwhile, distant vessels occupy few pixels, so a limited localization error may cause a substantial change in bounding-box overlap and weaken ReID feature discrimination. Sea-surface reflections and wave patterns may further affect both detection confidence and inter-frame image alignment. These coupled effects motivate a tracking framework that coordinates observation quality, state updating, camera-motion correction, and low-confidence association. For example, Shao et al. [19] proposed a maritime multi-object tracking method for autonomous ship navigation. By developing an on-board camera motion compensation module and designing a progressive refinement cascade matching strategy, the proposed method improved target tracking accuracy and stability in complex maritime environments. Hao et al. [20] proposed a lightweight ship multi-object tracking method based on trajectory association and an improved YOLOv7-tiny model. The feature extraction network was optimized during the detection stage, while an OSNet re-identification network and a noise scale adaptive (NSA) Kalman filtering algorithm were introduced during the tracking stage to reduce identity switches in ship tracking. Luo et al. [21] proposed the JDR-CSTrack ship multi-object tracking framework, which jointly models target appearance information and motion states to achieve multi-level data association. This method effectively reduces track loss and identity switches during the tracking process, providing a new technical perspective for ship multi-object tracking research. However, existing methods still mainly rely on appearance features and motion models for association. Under complex maritime conditions, they remain vulnerable to trajectory fragmentation and identity switches due to fluctuations in detection confidence, missed detections of small targets, and camera motion.

Although significant progress has been achieved in maritime target detection and tracking research in recent years, maintaining target identity consistency in complex maritime environments remains a considerable challenge. Unlike terrestrial traffic scenarios, maritime videos are affected by various factors, including dynamic sea backgrounds, illumination variations, wave disturbances, small-scale distant targets, and camera motion, which can easily lead to unstable motion prediction, failed target association, and frequent identity switches [22,23]. Target identity consistency is an essential foundation for reliable maritime perception. Continuous and stable target trajectories can provide critical information for ship behavior analysis, collision avoidance decision-making, autonomous navigation, and maritime monitoring. When frequent identity switches occur during the tracking process, the temporal information of individual ships becomes unreliable, thereby reducing the effectiveness of subsequent decision-making and behavior analysis. Therefore, developing a multi-object tracking method capable of maintaining high identity consistency in complex maritime environments is of great significance for the development of intelligent maritime systems [24,25].

This study develops an integrated ship detection and multi-object tracking framework, termed SeaTrack-DeepSORT, for maritime videos affected by small targets, uncertain detections, and camera motion. SeaDet-YOLO adapts the YOLOv11 detector by incorporating the existing ADown operation and an additional P2 detection branch for maritime small-target detection. The tracking module is built upon DeepSORT and combines confidence-dependent state updating, ECC-Affine camera-motion correction, and two-stage association. Therefore, the principal contribution of this work lies in the maritime-specific adaptation and coordinated integration of these components.

## 2. Background

### 2.1. YOLOv11 Architecture

The YOLO series performs object classification and bounding-box regression within a unified one-stage detection framework and has been widely used in real-time visual perception tasks [26]. YOLOv11 retains this one-stage structure and consists primarily of a backbone for hierarchical feature extraction, a neck for multi-scale feature fusion, and a decoupled detection head for classification and localization [27], as shown in Figure 1.

In this study, YOLOv11 is used as the detector baseline. The aspects relevant to the proposed modifications are its progressive downsampling operations and its original P3–P5 detection structure. The corresponding maritime-oriented adaptations are described in Section 3.1.

### 2.2. DeepSORT Framework

DeepSORT is a tracking-by-detection framework that receives object detections from an external detector and maintains target identities across consecutive frames [28]. It combines Kalman-filter motion prediction, ReID appearance features, cascade matching, and track management. Its overall workflow is illustrated in Figure 2.

Each DeepSORT track is represented by an eight-dimensional state containing the bounding-box center, aspect ratio, height, and their corresponding velocities. During association, the cosine distance between ReID features is used to measure appearance similarity, while the Mahalanobis distance is used to evaluate motion consistency between detections and predicted tracks [29]. The resulting assignment is solved using the Hungarian algorithm, after which matched tracks are updated and unmatched tracks are managed according to their lifecycle states [30].

In this study, the standard DeepSORT framework serves as the tracking baseline. Its basic tracking-by-detection procedure is retained, while the measurement update, camera-motion correction, and association process are adapted for maritime video.

## 3. Proposed Method

### 3.1. SeaDet-YOLO Detector

#### 3.1.1. SeaDet-YOLO Algorithm

In recent years, the YOLO series object detection algorithms have been widely applied in target detection tasks under complex environments due to their high detection accuracy and real-time performance. However, in practical maritime visual perception scenarios, ship targets usually exhibit significant scale variations, a high proportion of distant targets, and complex background interference. In particular, small-scale ship targets occupy only a limited number of pixels in images, making fine-grained information easily lost during feature extraction and downsampling processes, which leads to missed detections and degraded localization accuracy. Therefore, this study improves the YOLOv11 baseline network and proposes an enhanced detector, termed SeaDet-YOLO, for maritime ship detection. The overall architecture of the proposed method is shown in Figure 3.

The original YOLOv11 network extracts multi-scale feature information through progressive downsampling operations. Although this strategy effectively enhances high-level semantic feature representation, the continuous downsampling process reduces the spatial resolution of feature maps, resulting in the gradual loss of edge information and fine-grained texture details of small targets. To address the problems of weak features and significant scale variations in distant small-scale ships in complex maritime environments, an ADown downsampling module is incorporated into the backbone network to replace the original downsampling operation. By integrating average pooling and max pooling branches, ADown reduces the feature map size while preserving more structural information of targets. Compared with conventional convolution-based downsampling methods, ADown can effectively reduce feature information loss and improve the feature extraction capability of the network for ship targets in complex maritime environments [31]. Furthermore, considering the small size and limited pixel information of ship targets in maritime scenarios, an additional P2 small-object detection head is incorporated into the original P3–P5 detection structure of YOLOv11, forming a four-scale detection architecture from P2 to P5. By increasing the resolution of shallow feature maps, the resulting four-scale structure preserves more spatial details and enhances the perception capability of the model for distant and small-scale ship targets. Meanwhile, the multi-scale feature fusion mechanism fully integrates low-level spatial localization information and high-level semantic information, thereby improving ship detection accuracy under complex maritime conditions.

#### 3.1.2. ADown Module

In the original YOLOv11 network, feature downsampling is mainly achieved through convolution operations. Although convolution-based downsampling can effectively reduce the spatial resolution of feature maps and enlarge the receptive field, the continuous downsampling process may cause the loss of spatial details of small targets. For maritime ship detection tasks, distant ship targets usually occupy only a small number of pixels in the image, resulting in weak edge, texture, and local structural features. The conventional downsampling strategy may gradually weaken these effective features, thereby degrading the detection performance of small targets.

The existing ADown structure is adopted to replace selected downsampling convolutions in the SeaDet-YOLO backbone. As shown in Figure 4, the input first undergoes average pooling with a 2×2 kernel and a stride of 1, after which the feature channels are divided into two branches. One branch applies a 3×3 convolution with a stride of 2. The other applies max pooling with a 3×3 kernel and a stride of 2, followed by a 1×1 convolution. The branch outputs are concatenated to form the downsampled features. Within SeaDet-YOLO, this backbone configuration is combined with the P2 detection branch to support feature extraction and detection at different spatial scales.

Accordingly, ADown is treated as an adopted downsampling operation, while the work-specific adaptation is its placement in the YOLOv11 backbone and its combination with the additional P2 detection branch for maritime small-target detection.

#### 3.1.3. P2 Detection Head

In complex maritime environments, distant ship targets usually occupy only a small number of pixels in images, and their effective features are easily lost during the progressive downsampling process of deep neural networks. The original YOLOv11 network adopts a three-scale detection structure consisting of P3–P5 layers. Although this structure can effectively detect targets with different scales, the lowest detection scale is P3/8, resulting in relatively low feature map resolution and limited capability for localization and feature representation of small-scale ship targets. Therefore, an additional P2 small-object detection head is incorporated into SeaDet-YOLO to construct a four-scale detection structure from P2 to P5, thereby enhancing the detection capability of the model for small ship targets in maritime environments.

The P2 detection head introduces higher-resolution shallow feature maps, enabling the network to preserve more spatial details of targets. Compared with the P3 feature layer, the P2 feature map undergoes fewer downsampling operations and therefore maintains higher spatial resolution, which can effectively preserve edge information, texture details, and local structural features of small targets. Meanwhile, the P2 layer is fused with deep semantic features, allowing the detection head to achieve both strong localization capability and semantic representation ability. Specifically, an additional feature upsampling path is introduced into the Neck of YOLOv11, where high-level semantic features are progressively restored to higher spatial resolutions and fused with the shallow P2 features from the Backbone. The fused features are further refined through the C3k2 module, ultimately forming the P2 small-object detection branch. By constructing a four-scale detection architecture from P2 to P5, the P2 layer is responsible for detecting small-scale ship targets, while the P3–P5 layers are utilized for detecting targets at different scales. This multi-scale detection structure enables the model to fully exploit hierarchical feature information, thereby enhancing the perception capability for small-scale ship targets under complex maritime conditions and improving detection accuracy.

The work-specific modification is the incorporation of the P2 branch into YOLOv11 and its coordination with the ADown-based backbone, rather than the proposal of a new generic detection-head mechanism.

### 3.2. SeaTrack-DeepSORT Tracker

#### 3.2.1. NSA Kalman Filtering

DeepSORT employs a Kalman filter to estimate the motion state of each target. Its state-space model can be expressed as

(1) $$x_{k}=Fx_{k-1}+w_{k},$$

where $x_{k}$ denotes the target state vector at frame *k*, *F* is the state–transition matrix, and $w_{k}$ denotes the process noise, which is assumed to follow a zero-mean Gaussian distribution with covariance $Q_{k}$.

Given a detection associated with an existing track, the observation model is expressed as

(2) $$z_{k}=Hx_{k}+v_{k}$$

where $z_{k}$ denotes the detection measurement, *H* is the observation matrix, and $v_{k}$ represents the measurement noise with covariance $R_{k}$.

In the original DeepSORT implementation, the baseline measurement-noise covariance is constructed according to the scale of the predicted bounding box. This design accounts for target-scale variation but does not explicitly incorporate the confidence of the matched detection. In maritime videos, detection reliability may vary because of small target size, sea-surface interference, illumination changes, partial occlusion, and image motion. Consequently, detections with different confidence scores should not necessarily contribute equally to the state update.

Let $R_{k}^{\mathrm{DS}}$ denote the baseline measurement-noise covariance generated by DeepSORT for the predicted state at frame *k*. After a detection has been associated with an existing track, its confidence score is used to scale this covariance as follows:

(3) $$R_{k}=\lambda_{k}R_{k}^{\mathrm{DS}}$$

where $\lambda_{k}$ is the confidence-dependent scaling factor that controls the contribution of the matched observation to the state update. The quadratic scaling function used in this study is defined as

(4) $$\lambda_{k}={\left(1-\alpha c_{k}\right)}^{2}$$

where $c_{k}\in \left[0,1\right]$ denotes the confidence score of the matched detection and 0<α<1 is a fixed coefficient controlling the range of the covariance adjustment. The mathematical behavior of the scaling function is given by

(5) $$\frac{\partial \lambda_{k}}{\partial c_{k}}=-2\alpha \left(1-\alpha c_{k}\right)<0,\;{\left(1-\alpha \right)}^{2}\leq \lambda_{k}\leq 1$$

The positive scalar $\lambda_{k}$ is introduced to adjust the overall magnitude of the baseline covariance $R_{k}^{\mathrm{DS}}$ without changing its relative covariance structure. Because $\lambda_{k}>0$, the scaled covariance remains positive semidefinite. The quadratic form ${\left(1-\alpha c_{k}\right)}^{2}$ provides a smooth, monotonic, and bounded mapping from detection confidence to measurement uncertainty. When $c_{k}=0$, $\lambda_{k}=1$, and the original DeepSORT covariance is retained. As $c_{k}$ increases, $\lambda_{k}$ decreases, allowing the matched observation to receive greater weight during the Kalman update. Moreover, the magnitude of the derivative gradually decreases with increasing confidence, which avoids an abrupt reduction in covariance for already high-confidence detections.

The restriction $\lambda_{k}\leq 1$ is deliberate. In this design, $R_{k}^{\mathrm{DS}}$ is treated as the reference uncertainty supplied by the original DeepSORT model, and detection confidence is used only to strengthen the influence of reliable matched observations rather than to inflate the reference covariance. Low-confidence detections are already handled conservatively by the confidence-based partition and the second association stage, and they are not used to initialize new tracks. Allowing $\lambda_{k}>1$ would further weaken the correction from these observations and could excessively suppress useful measurements. Therefore, the mapping is intended as a one-sided reliability weighting rather than an absolute calibration of measurement noise.

In all experiments, α was fixed at 0.3, giving $0.49\leq \lambda_{k}\leq 1$. In the limiting case α=0, the method reduces to the original DeepSORT covariance because $\lambda_{k}=1$. Small positive values of α produce conservative adjustments, whereas larger values increase the influence of detection confidence on the Kalman update. For example, α=0.1, 0.3, and 0.5 correspond to minimum scaling factors of 0.81, 0.49, and 0.25, respectively. Values close to one may cause the filter to over-trust high-confidence detections. Thus, α=0.3 was used as a conservative setting and was kept unchanged across all tracking experiments.

More specifically, the Kalman gain is computed as

(6) $$K_{k}=P_{k}^{-}H^{T}{\left(HP_{k}^{-}H^{T}+R_{k}\right)}^{-1}$$

where $P_{k}^{-}$ is the predicted state covariance. Therefore, decreasing $R_{k}$ for a high-confidence detection generally increases the contribution of that observation to the corrected state. In contrast, a low-confidence detection retains a covariance closer to the DeepSORT baseline and therefore has a relatively smaller influence on the state correction.

The resulting confidence-aware noise adjustment dynamically scales the measurement-noise covariance according to the confidence of the matched detection. High-confidence detections are assigned a smaller measurement covariance, allowing the Kalman filter to place greater weight on reliable observations, whereas low-confidence detections retain a larger covariance to reduce their influence on the state update. By introducing the coefficient α and adopting a quadratic confidence mapping, the adjustment range can be controlled while avoiding an excessive reduction in measurement uncertainty for high-confidence detections.

#### 3.2.2. Two-Stage Association

As shown in Figure 5, confidence-based detection partitioning is incorporated into the DeepSORT association pipeline. High-confidence detections are first matched with existing tracks using motion and appearance information. Tracks that remain unmatched are then considered for recovery using low-confidence detections. This separation allows detections with reduced confidence to remain available for track continuation while excluding them from new-track initialization. In maritime video, the recovery stage is intended to retain potentially useful observations when partial occlusion, small target size, or background interference reduces detection confidence.

In the first stage, high-confidence detections are associated with existing tracks using the DeepSORT matching cascade. For confirmed tracks, the cosine distance between the ReID appearance features is used as the association cost, while the motion information predicted by the nonlinear NSA Kalman filter is used as a gating constraint. Specifically, a detection–track pair is rejected when its squared Mahalanobis distance exceeds the gating threshold. The maximum cosine distance is set to 0.2, and the Mahalanobis gating threshold is set to 9.4877, corresponding to the 95% quantile of a chi-square distribution with four degrees of freedom. The Hungarian algorithm is then applied to the gated appearance–cost matrix. Remaining unconfirmed tracks and recently unmatched confirmed tracks are further associated using IoU matching, for which the maximum IoU distance is set to 0.7, corresponding to a minimum acceptable IoU of 0.3. Therefore, motion and appearance costs are not combined through fixed linear association weights in the implementation; instead, appearance distance is used for assignment and motion consistency is enforced through Mahalanobis gating. Successfully associated tracks are updated directly, whereas the remaining unmatched tracks are retained for recovery in the second association stage.

The detector outputs are divided into high- and low-confidence sets using two thresholds. Detections with $c_{k}\geq \tau_{\mathrm{high}}$ are assigned to the high-confidence set, whereas detections satisfying $\tau_{\mathrm{low}}\leq c_{k}<\tau_{\mathrm{high}}$ are assigned to the low-confidence set. In all tracking experiments, $\tau_{\mathrm{high}}=0.5$ and $\tau_{\mathrm{low}}=0.1$.

For the trajectories that remain unmatched in the first stage, a second association stage using low-confidence detections is further incorporated. In this stage, the predicted target positions of unmatched trajectories obtained from the Kalman filter are compared with low-confidence detection boxes by calculating the Intersection over Union (IoU). An IoU-based matching matrix is then constructed:

(7) $$IoU\left(B_{i},B_{j}\right)=\frac{|B_{i}\cap B_{j}|}{|B_{i}\cup B_{j}|}$$

where $B_{i}$ represents the predicted bounding box of the trajectory, and $B_{j}$ denotes the low-confidence detection bounding box. Subsequently, the Hungarian algorithm based on IoU is employed to complete the matching process. Through the above two-stage association process, trajectory interruptions caused by short-term occlusion, target ambiguity, or decreased detection confidence can be effectively recovered. It should be noted that low-confidence detections are only utilized for recovering existing trajectories and are not involved in new track initialization, thereby avoiding the introduction of incorrect identity information caused by unreliable detections. After completing the two-stage data association, both successfully matched trajectories and recovered trajectories are used for target state updates. Subsequently, the trajectory management module is employed to create new tracks and remove invalid trajectories. The integrated two-stage association procedure utilizes the potential useful information contained in low-confidence detections. While maintaining the advantages of DeepSORT in appearance feature matching, it further improves the trajectory continuity and robustness of ship tracking in complex maritime environments.

#### 3.2.3. Camera Motion Compensation

In vision-based tracking tasks for unmanned surface vehicles, the captured video sequences may contain considerable camera motion caused by platform movement, wave disturbances, and vibrations of the camera mounting structure. Unlike fixed-camera scenarios, changes in vessel attitude produce inter-frame viewpoint shifts and cause targets to exhibit similar global displacements in the image plane. Consequently, the apparent motion of a target contains both its own motion and the displacement induced by the imaging platform. If this global displacement is not considered, the motion prediction of DeepSORT may become spatially inconsistent with the detections in the current frame, increasing the difficulty of data association.

To reduce this inconsistency, an Enhanced Correlation Coefficient (ECC)-based camera-motion compensation module is employed in SeaTrack-DeepSORT. ECC estimates a parametric transformation between two consecutive frames by optimizing an image-level correlation criterion. Unlike feature point matching-based approaches, it does not require explicit keypoint detection and matching. This property makes ECC applicable when stable local feature points are difficult to obtain. Nevertheless, its estimation quality may still be affected by low-texture regions, moving foreground targets, illumination changes, and large inter-frame displacements. Therefore, ECC is used in this study as an approximate inter-frame alignment method rather than as an exact physical estimate of camera motion.

The ECC algorithm estimates the transformation parameters between consecutive frames by maximizing the enhanced correlation coefficient between a reference image and a geometrically transformed image [32]. The corresponding normalized registration error is expressed as

(8) $$E_{\mathrm{ECC}}\left(p\right)={∥\frac{{\hat{i}}_{r}}{∥{\hat{i}}_{r}∥}-\frac{{\hat{i}}_{w}\left(p\right)}{∥{\hat{i}}_{w}\left(p\right)∥}∥}^{2},$$

where *p* denotes the transformation parameters, ${\hat{i}}_{r}$ is the zero-mean pixel vector of the reference image, and ${\hat{i}}_{w}\left(p\right)$ is the zero-mean pixel vector obtained after warping the adjacent frame according to *p*. The enhanced correlation coefficient is defined as

(9) $$\rho \left(p\right)=\frac{{\hat{i}}_{r}^{T}{\hat{i}}_{w}\left(p\right)}{∥{\hat{i}}_{r}∥∥{\hat{i}}_{w}\left(p\right)∥},$$

where a larger value of ρ(p) indicates greater consistency between the reference image and the warped image. By optimizing this criterion, ECC obtains an inter-frame geometric transformation that approximates the global image displacement.

Considering that consecutive maritime video frames may contain translation, small rotation, scale variation, and shear, an affine transformation is used to describe the inter-frame image displacement:

(10) $$\left[\begin{array}{c} x^{\prime }\\ y^{\prime } \end{array}\right]=\left[\begin{array}{cc} a_{11} & a_{12}\\ a_{21} & a_{22} \end{array}\right]\left[\begin{array}{c} x\\ y \end{array}\right]+\left[\begin{array}{c} t_{x}\\ t_{y} \end{array}\right].$$

Here, (x,y) and $\left(x^{\prime },y^{\prime }\right)$ denote the point coordinates before and after inter-frame alignment, respectively. The coefficients $a_{11}$, $a_{12}$, $a_{21}$, and $a_{22}$ constitute the linear component of the affine transformation, while $t_{x}$ and $t_{y}$ represent horizontal and vertical translation. The direction of the transformation is set so that the compensated track coordinates and the detections are expressed in the coordinate system of the current frame.

In SeaTrack-DeepSORT, the ECC-Affine module is inserted between Kalman prediction and data association. Let $p_{k}^{-}={\left[u_{k}^{-},v_{k}^{-}\right]}^{T}$ denote the center of the predicted bounding box at frame *k*. The ECC-compensated center used for data association is calculated as

(11) $$p_{k}^{\mathrm{ECC}}=\left[\begin{array}{cc} a_{11} & a_{12}\\ a_{21} & a_{22} \end{array}\right]p_{k}^{-}+\left[\begin{array}{c} t_{x}\\ t_{y} \end{array}\right].$$

where $p_{k}^{\mathrm{ECC}}={\left[u_{k}^{\mathrm{ECC}},v_{k}^{\mathrm{ECC}}\right]}^{T}$ denotes the compensated center in the coordinate system of the current frame. During data association, the compensated predicted state is used for Mahalanobis gating, while the corresponding bounding box is used for IoU computation.

The compensation is restricted to the predicted center coordinates. The aspect-ratio, height, and velocity components of the Kalman state are kept unchanged; consequently, the derived bounding-box width is also unchanged. The compensated center is directly written back to the position components of the predicted Kalman state mean before data association. After data association, a successfully matched track is updated using the corresponding detection through the standard Kalman measurement-update procedure. Therefore, the ECC-Affine module is implemented as a position-only correction of the predicted Kalman state mean rather than as a complete affine transformation of the Kalman state distribution.

Although the compensated center is written back to the position components of the predicted Kalman state mean, the Kalman covariance matrix and its cross-covariance terms are not explicitly transformed by the affine matrix. This position-only approximation assumes that the inter-frame camera motion is moderate and that changes in target size and shape between adjacent frames are limited. Although the affine model can represent rotation, scaling, translation, and shear at the image level, the present implementation uses this transformation only to correct the predicted center. It should therefore not be interpreted as full-state geometric compensation. Under large rotations, abrupt zooming, or strong perspective changes, center-only correction may not fully describe the effect of camera motion on the target state.

The compensation is applied only to the predicted target center, while the other state variables, such as target size and velocity, remain unchanged. The compensated position is directly written back to the position components of the predicted Kalman state mean before subsequent data association. This design reduces the influence of camera-motion estimation errors on the original motion model while improving the spatial consistency between predicted trajectories and current-frame detections.

The resulting ECC-compensated boxes are used in the subsequent association stages to reduce the spatial mismatch between predicted track centers and current-frame detections. Within the complete tracking pipeline, NSA Kalman filtering controls the confidence-dependent measurement update, ECC-Affine provides association-level center correction, and the two-stage association strategy recovers existing trajectories using low-confidence detections. These components operate at different stages of the tracking process and jointly improve track association and trajectory continuity under the evaluated maritime conditions.

### 3.3. Integrated Detection and Tracking Framework

The integrative contribution of SeaTrack-DeepSORT lies in coordinating multiscale detection, confidence-dependent state updating, association-level camera-motion correction, and low-confidence track recovery within a unified maritime tracking pipeline. Although object detection algorithms can obtain the position and category information of ships in individual image frames, they cannot guarantee identity consistency across consecutive video frames due to the lack of temporal association between detection results. Therefore, this study combines the improved SeaDet-YOLO with SeaTrack-DeepSORT to construct a multi-object detection and tracking framework for complex maritime environments, enabling continuous localization and identity preservation of ship targets in dynamic water scenes.

In the proposed framework, SeaDet-YOLO serves as the front-end visual detection module, which performs ship detection on each frame of the input video sequence and outputs the bounding box locations, category information, and confidence scores of detected ships. To address the challenges of significant target scale variations, weak features of small-scale ships, and complex background interference in maritime environments, SeaDet-YOLO incorporates the ADown downsampling module and an additional P2 small-object detection head to enhance feature extraction capability for ships with different scales, providing more accurate and reliable observation information for the subsequent tracking process.

After obtaining the detection results, SeaTrack-DeepSORT is employed as the back-end multi-object tracking module to perform cross-frame association and trajectory maintenance. First, the NSA-based Kalman filter predicts the future motion state of targets based on existing trajectory information, while the ECC-Affine camera-motion compensation module is employed to reduce the global displacement caused by the motion of the unmanned surface vehicle platform. Subsequently, joint association is performed by integrating target appearance features and motion information. Furthermore, the two-stage association strategy exploits low-confidence detections to recover potentially lost trajectories, thereby improving trajectory continuity and identity preservation under complex maritime conditions.

The overall detection-tracking pipeline is illustrated in Figure 6. For each input frame, SeaDet-YOLO first performs ship detection and generates candidate ship targets. The detection results are then fed into SeaTrack-DeepSORT for data association with existing trajectories. For successfully matched detections, the corresponding trajectory states are updated while maintaining their original identity numbers. For unmatched new targets that satisfy the initialization criteria, new trajectories are created. For targets temporarily missing due to short-term detection failure, the trajectory management strategy preserves them for a certain period and enables subsequent recovery using future detection results. As the video sequence continuously arrives, the above process is iteratively executed to achieve continuous detection, localization, and identity preservation of ship targets in complex maritime environments.

The framework connects detection, state updating, and association through the bounding boxes, confidence scores, and predicted track states passed between stages. SeaDet-YOLO provides the detection observations, while ECC-Affine corrects predicted target centers before association to account for inter-frame camera motion. Detection confidence has two roles: it determines whether a detection participates in the first association stage or the recovery stage, and it scales the measurement noise when the matched track is updated. Low-confidence detections can therefore support existing tracks without initiating new identities. These operations form an integrated ship detection and tracking pipeline for handling observation variability and platform-induced displacement.

## 4. Experiments and Analysis

### 4.1. Datasets

In this study, the Water Surface Object Detection Dataset (WSODD), proposed by Zhou et al. [33], is used to train and evaluate the SeaDet-YOLO detector. WSODD contains 7467 water-surface images with 21,911 annotated instances from 14 object categories, including ships, buoys, and persons. The images were collected in oceans, lakes, and rivers under different weather and illumination conditions, including sunny, cloudy, foggy, daytime, twilight, and nighttime scenes. As illustrated in Figure 7, the dataset includes variations in background complexity, target visibility, and object scale, which are representative of the visual challenges encountered in water-surface object detection.

The 7467 images were divided at the image level into training, validation, and test sets, containing 5226, 1493, and 748 images, respectively, corresponding to an approximate ratio of 70%:20%:10%. All 14 object categories were retained during training and evaluation. The training set was used for model optimization, the validation set was used to monitor convergence and select the final model checkpoint, and the test set was used to report the detection results. The same dataset split was used for all compared detectors and all ablation configurations to ensure that the reported results were obtained under consistent experimental conditions.

In this study, the ShipsMOT (Ships Multi-Object Tracking Dataset) is adopted as the experimental dataset for maritime multi-object tracking. ShipsMOT is a dedicated dataset developed for ship visual perception tasks, aiming to facilitate research on ship detection, multi-object tracking, and intelligent maritime perception algorithms in complex marine environments. The dataset is collected from real-world maritime video sequences and provides continuous video frames, target bounding boxes, and corresponding identity annotations, effectively reflecting the dynamic variations in ship targets in practical maritime scenarios.

The ShipsMOT dataset contains multiple real-world maritime video sequences, covering ship targets with different viewpoints, scales, and motion states. Compared with conventional single-frame object detection datasets, ShipsMOT provides target bounding boxes and identity IDs across consecutive video frames, enabling comprehensive evaluation of multi-object tracking algorithms in terms of target association, trajectory continuity, and identity preservation. The dataset includes typical challenges in maritime environments, such as ship scale variations, target occlusions, target interactions, and complex sea surface backgrounds, which can effectively simulate the challenges encountered by USV-based visual perception systems during practical operation.

Due to the high appearance similarity of ship targets, significant variations in motion states, and susceptibility to camera motion in maritime environments, conventional multi-object tracking algorithms often suffer from trajectory fragmentation and identity switches under complex sea conditions. Therefore, ShipsMOT provides an effective benchmark for evaluating the robustness of tracking algorithms in realistic maritime scenarios. In this study, the proposed SeaTrack-DeepSORT framework is evaluated on the ShipsMOT dataset. Given continuous video sequences, SeaDet-YOLO first provides ship detection results, and the proposed tracking framework integrates NSA Kalman filtering, ECC-Affine camera motion compensation, and the two-stage association strategy to achieve continuous ship tracking and identity preservation in complex maritime environments.

### 4.2. Experimental Setup

The experiments were conducted on a workstation running the Windows 11 Professional operating system. The platform was equipped with two Intel Xeon Gold 6226R processors (Intel Corporation, Santa Clara, CA, USA), an NVIDIA RTX 5880 Ada GPU (NVIDIA Corporation, Santa Clara, CA, USA), and 48 GB of memory. The models were implemented in Python 3.12 using the PyTorch 2.5.1 framework, with CUDA 12.4 used for GPU acceleration.

For detector training, stochastic gradient descent (SGD) was employed as the optimizer. The models were trained for 200 epochs with an input resolution of 640×640 pixels and a batch size of 32. The momentum and weight-decay coefficients were set to 0.937 and 0.0005, respectively. The initial learning rate was set to 0.01, and the final learning-rate factor (lrf) was set to 0.01.

The principal parameters of SeaTrack-DeepSORT are summarized in Table 1. Unless otherwise specified, these parameters were kept fixed in all experiments involving SeaTrack-DeepSORT.

Fixed association weights are not used in the implemented tracker. During first-stage association, the cosine distance between ReID appearance features is used as the assignment cost, while the motion prediction produced by the Kalman filter is incorporated through Mahalanobis gating. The Mahalanobis gating threshold of 9.4877 corresponds to the 95% quantile of a chi-square distribution with four degrees of freedom. Remaining eligible tracks are associated using IoU matching.

A newly initialized track remains tentative until it is successfully detected in three consecutive frames. A tentative track is deleted if it fails to obtain a match before confirmation. A confirmed track is retained during short-term missed detections and is deleted when the number of consecutive missed frames exceeds 70. Low-confidence detections are used only to recover existing unmatched tracks and are not used to initialize new tracks.

### 4.3. Evaluation Metrics

To comprehensively evaluate the performance of the proposed SeaDet-YOLO and SeaTrack-DeepSORT joint framework in maritime ship detection and multi-object tracking tasks, this study conducts quantitative analyses from two aspects: object detection accuracy and tracking performance. In the object detection stage, Precision (P), Recall (R), F1-score, and mean Average Precision (mAP) are adopted to evaluate the ship detection performance of SeaDet-YOLO. In the multi-object tracking stage, Higher Order Tracking Accuracy (HOTA), Multi-Object Tracking Accuracy (MOTA), Identity F1 Score (IDF1), False Positives (FP), False Negatives (FN), and Identity Switches (IDSW) are employed to evaluate the tracking performance of SeaTrack-DeepSORT.

Precision is used to measure the reliability of detection results, representing the proportion of correctly predicted targets among all detected targets. It is calculated as follows:

(12) $$P=\frac{TP}{TP+FP}$$

where TP (True Positive) represents the number of correctly detected targets, and FP (False Positive) represents the number of incorrectly detected targets. A higher Precision indicates that the model can effectively reduce false detections caused by background interference.

Recall (R) is used to measure the completeness of target detection, representing the proportion of correctly detected targets among all actual targets. It is calculated as follows:

(13) $$R=\frac{TP}{TP+FN}$$

where FN (False Negative) represents the number of missed targets. In maritime environments, target omissions are easily caused by scale variations, target occlusions, and complex background interference. Therefore, Recall can reflect the detection model’s ability to perceive target objects completely.

Since Precision and Recall focus on false detections and missed detections, respectively, this study further adopts the F1-score to provide a comprehensive evaluation of both metrics:

(14) $$F1=\frac{2PR}{P+R}$$

where F1-score considers the effects of both Precision and Recall and can provide a more comprehensive evaluation of the model detection performance.

Average Precision (AP) is a commonly used evaluation metric in object detection tasks, which measures the matching performance between predicted bounding boxes and ground-truth annotations. AP is obtained by calculating the area under the Precision–Recall (P-R) curve, and its definition is given as follows:

(15) $$AP=\int_{0}^{1}P\left(R\right)dR$$

where *P* represents the Precision under different confidence thresholds, and *R* represents the corresponding Recall values. A higher AP value indicates that the model achieves better target localization and classification performance under different detection thresholds.

For multi-class object detection tasks, mean Average Precision (mAP) is adopted to comprehensively evaluate the detection performance of all categories. It is calculated as follows:

(16) $$mAP=\frac{1}{N}\sum_{i=1}^{N}AP_{i}$$

where *N* represents the number of target categories, and $AP_{i}$ denotes the Average Precision of the *i*-th category.

In the multi-object tracking task, this study adopts Higher Order Tracking Accuracy (HOTA), Multi-Object Tracking Accuracy (MOTA), and Identity F1 Score (IDF1) as the primary evaluation metrics. In addition, False Positive (FP), False Negative (FN), and Identity Switches (IDSW) are also calculated to evaluate the tracking performance of the proposed algorithm in complex maritime environments.

Multi-Object Tracking Accuracy (MOTA) is used to comprehensively evaluate tracking errors caused by missed detections, false detections, and identity switches during the tracking process. It is calculated as follows:

(17) $$MOTA=1-\frac{\sum_{t}\left(FN_{t}+FP_{t}+{\mathrm{IDSW}}_{t}\right)}{\sum_{t}GT_{t}}$$

where $FN_{t}$ represents the number of missed targets in frame *t*, $FP_{t}$ represents the number of false detections, ${\mathrm{IDSW}}_{t}$ represents the number of identity switches, and $GT_{t}$ represents the number of ground-truth targets in frame *t*. A higher MOTA value indicates that the tracking algorithm achieves better overall performance in terms of detection accuracy and identity preservation.

Higher Order Tracking Accuracy (HOTA) is a comprehensive evaluation metric that simultaneously considers target detection accuracy, association accuracy, and trajectory localization quality. It is calculated as follows:

(18) $$\mathrm{HOTA}=\frac{1}{19}\sum_{\alpha \in \{0.05,0.10,\ldots ,0.95\}}\sqrt{\frac{\sum_{c\in \{{\mathrm{TP}}_{\alpha }\}}A_{\alpha }\left(c\right)}{|{\mathrm{TP}}_{\alpha }\left|+\right|{\mathrm{FN}}_{\alpha }\left|+\right|{\mathrm{FP}}_{\alpha }|}}$$

where α denotes the localization threshold; ${\mathrm{TP}}_{\alpha }$, ${\mathrm{FN}}_{\alpha }$, and ${\mathrm{FP}}_{\alpha }$ denote the sets of true-positive matches, false-negative detections, and false-positive detections at threshold α, respectively; and $A_{\alpha }\left(c\right)$ denotes the association accuracy of the true-positive match *c*. The final HOTA score is obtained by averaging the results over 19 localization thresholds ranging from 0.05 to 0.95 at intervals of 0.05. A higher HOTA value indicates better overall detection, association, and localization performance.

All tracking metrics were computed using the TrackEval toolkit with the same ground-truth annotations and evaluated frame ranges. Following the standard TrackEval protocol, the final HOTA score was averaged over 19 localization thresholds ranging from 0.05 to 0.95 at intervals of 0.05.

Identity F1 Score (IDF1) is used to evaluate the accuracy of target identity association during the tracking process. This metric is obtained by calculating the harmonic mean of Identity Precision (IDP) and Identity Recall (IDR).

Identity Precision is defined as:

(19) $$IDP=\frac{TP_{id}}{TP_{id}+FP_{id}}$$

Identity Recall is defined as:

(20) $$IDR=\frac{TP_{id}}{TP_{id}+FN_{id}}$$

The IDF1 score is further calculated as:

(21) $$IDF1=\frac{2TP_{id}}{2TP_{id}+FP_{id}+FN_{id}}$$

where $TP_{id}$ represents the number of correctly matched identities, $FP_{id}$ represents the number of incorrectly matched identities, and $FN_{id}$ represents the number of lost identities. A higher IDF1 value indicates stronger capability of maintaining target identity consistency during continuous tracking.

Furthermore, FP, FN, and IDSW are reported as auxiliary error counts. FP denotes the total number of predicted detections that are not matched to any ground-truth target over the evaluated frames, whereas FN denotes the total number of ground-truth detections that are not matched by any prediction. IDSW counts the events in which the predicted track identity assigned to a ground-truth trajectory changes during evaluation. These metrics characterize different sources of tracking error and should not be interpreted as numbers of complete trajectories.

### 4.4. Results and Discussion

#### 4.4.1. Ship Detection Results

The proposed SeaDet-YOLO model is first evaluated in terms of object detection performance and compared with various mainstream object detection algorithms. The comparative experiments include the single-stage detector SSD, the two-stage detector Faster R-CNN, and several lightweight YOLO-series models, including YOLOv5n, YOLOv6n, YOLOv8n, YOLOv10n, and YOLOv11n. By comparing Precision (P), Recall (R), F1-score, mAP_0.5_, mAP_0.5:0.95_, the number of parameters (Params), and computational complexity (GFLOPs) on the WSODD dataset, the performance of different detection models in water surface object detection tasks is comprehensively evaluated. The experimental results are presented in Table 2.

SeaDet-YOLO achieves superior detection performance on the WSODD dataset. Compared with the conventional single-stage detector SSD, SeaDet-YOLO improves mAP_0.5_ and mAP_0.5:0.95_ by 18.57 percentage points and 18.42 percentage points, respectively. Meanwhile, the number of model parameters is reduced from 26.3 M to 2.3 M, with a significant reduction in computational complexity. These results indicate that the proposed method can achieve effective model lightweighting while maintaining high detection accuracy. Compared with the two-stage detector Faster R-CNN, SeaDet-YOLO improves mAP_0.5_ and mAP_0.5:0.95_ by 19.67 percentage points and 20.62 percentage points, respectively, while requiring considerably fewer parameters and lower computational cost. This demonstrates that the proposed method achieves a better trade-off among detection accuracy, computational efficiency, and practical deployment capability.

Further comparison with lightweight YOLO-series models demonstrates that SeaDet-YOLO achieves significant improvements over the baseline YOLOv11n model. Specifically, the Precision, Recall, F1-score, mAP_0.5_, and mAP_0.5:0.95_ are improved by 3.62%, 3.46%, 3.54%, 3.76%, and 2.95%, respectively. Although the computational complexity of SeaDet-YOLO is slightly increased compared with YOLOv11n, the model only contains 2.3 M parameters, indicating that the proposed method can improve detection accuracy while maintaining low model complexity. Therefore, it is suitable for real-time and lightweight deployment in USV-based visual perception systems.

Ablation experiments based on YOLOv11n are used to examine the contribution of each detector component, and the experimental results are presented in Table 3.

Introducing the ADown module improves the detection performance of the model. Compared with the original YOLOv11n, YOLOv11n + ADown increases mAP_0.5_ from 76.51% to 76.94% and improves mAP_0.5:0.95_ from 43.07% to 44.03%. Meanwhile, the number of parameters is reduced from 2.6 M to 2.2 M, and the GFLOPs decrease from 6.5 to 5.6, indicating that ADown improves feature representation while reducing model complexity. This improvement is mainly attributed to the optimized downsampling process of ADown, which reduces computational cost while preserving more effective spatial information, enabling the network to extract richer target features.

After introducing the P2 detection head, the detection performance is further improved. Compared with the original YOLOv11n, YOLOv11n + P2 improves mAP_0.5_ by 2.39 percentage points and mAP_0.5:0.95_ by 1.46 percentage points, demonstrating that the additional high-resolution detection layer effectively enhances the small-target detection performance. Since the WSODD dataset contains numerous distant and small water surface targets, the conventional P3–P5 detection structure may lose fine-grained spatial information during successive downsampling operations. In contrast, the P2 detection head introduces higher-resolution shallow features, preserving more target edge and texture information and thereby improving small-object detection performance.

The combined SeaDet-YOLO configuration achieves the highest detection accuracy in the ablation study. Compared with YOLOv11n, it increases mAP_0.5_ and mAP_0.5:0.95_ by 3.76 and 2.95 percentage points, respectively. The results show that ADown and the P2 detection head both contribute to the final detector performance under the evaluated configuration.

#### 4.4.2. Multi-Object Tracking Results

The proposed SeaTrack-DeepSORT multi-object tracking algorithm is further evaluated on selected sequences from the ShipsMOT dataset. The sequence selection was based on camera viewpoint and platform-motion characteristics, with the purpose of conducting a scenario-based comparison rather than estimating aggregate performance over the complete benchmark. Specifically, sequences 46 and 73 were selected to represent two contrasting camera configurations relevant to maritime tracking.

Sequence 46 was captured using a camera mounted on a moving vessel and therefore contains platform-induced camera motion, viewpoint variation, and relative target displacement. It was selected to evaluate the influence of the detector and the tracking components under an on-board moving-camera condition. In contrast, sequence 73 was captured using a fixed-position camera and was selected as a fixed-view case with relatively stable observation conditions. The comparison between these two sequences therefore examines the tracking framework under both moving-camera and fixed-camera conditions.

To investigate the influence of the detection module on multi-object tracking performance, YOLOv11 + DeepSORT is first adopted as the baseline method, and the original detector is replaced with the proposed SeaDet-YOLO to construct the SeaDet-YOLO + DeepSORT framework for comparison. Both methods employ the same DeepSORT tracking framework, with only the detection module being changed to ensure a fair comparison. The tracking performance is comprehensively evaluated using HOTA, MOTA and IDSW metrics, and the experimental results are presented in Table 4.

Compared with the YOLOv11 + DeepSORT method, the introduction of the proposed SeaDet-YOLO detector significantly improves multi-object tracking performance. For the on-board view video sequence 46, the camera platform is in a moving state, and factors such as vessel motion, camera disturbances, and target scale variations increase the difficulty of target tracking. The YOLOv11 + DeepSORT method achieves MOTA and HOTA values of 34.40% and 41.34%, respectively, with 13 identity switches. After replacing the detector with SeaDet-YOLO, the MOTA and HOTA are improved to 47.27% and 53.33%, respectively, while the number of identity switches is reduced to 0. These results indicate that more accurate detection results can provide more reliable observations for the tracker, thereby improving trajectory continuity and identity preservation in complex motion scenarios.

For the fixed-view video sequence 73, the influence of camera motion is relatively limited, and YOLOv11 + DeepSORT already achieves satisfactory tracking performance. On this basis, SeaDet-YOLO further improves MOTA from 96.24% to 98.23% and HOTA from 87.90% to 90.40%, while reducing identity switches. According to the average results of the two sequences, SeaDet-YOLO + DeepSORT improves the average MOTA from 65.32% to 72.75% and the average HOTA from 64.62% to 71.87%, while reducing IDSW from 13 to 2.5. These results demonstrate that SeaDet-YOLO enhances ship detection performance in complex water surface environments and provides more accurate target localization information for subsequent trajectory association, thereby improving the overall multi-object tracking performance.

To examine the performance changes obtained when the tracking components are introduced in their pipeline order, ShipsMOT sequence 46 is used for the progressive configuration comparison. This sequence was selected because its on-board camera contains platform-induced motion, viewpoint variation, and relative target displacement, which are directly related to the maritime tracking problems addressed by the proposed components. SeaDet-YOLO + DeepSORT is used as the baseline configuration, after which NSA Kalman filtering, ECC-Affine center correction, and two-stage association are added sequentially while the detection results remain unchanged. This experiment therefore reports incremental performance changes under a fixed integration order on sequence 46, rather than independent component effects or complete-benchmark performance. HOTA, MOTA, IDF1, FP, and FN are used as the evaluation metrics, and the results are presented in Table 5.

Incorporating NSA Kalman filtering produces consistent changes across the evaluated metrics. HOTA, MOTA, and IDF1 increase from 53.33%, 47.27%, and 71.15% to 53.46%, 47.37%, and 71.17%, respectively. Meanwhile, FP decreases from 338 to 335, while FN remains unchanged at 666. This result indicates that the confidence-dependent measurement-noise adjustment refines the state-update process without increasing missed targets. Within the complete framework, NSA Kalman filtering serves as a conservative state-estimation component that regulates the contribution of detection observations and provides a stable basis for subsequent motion compensation and association.

After ECC-Affine center correction is introduced, HOTA, MOTA, and IDF1 reach 53.47%, 47.90%, and 71.46%, respectively. FP decreases from 335 to 330, while FN decreases from 666 to 662. These changes are consistent with the intended function of ECC-Affine in reducing the spatial mismatch between predicted track centers and current-frame detections. However, the aggregate metrics do not independently demonstrate that camera-induced displacement is completely eliminated.

The largest incremental improvement occurs after introducing the two-stage association strategy. Compared with the preceding configuration, HOTA increases from 53.47% to 55.00%, MOTA increases from 47.90% to 54.31%, and IDF1 increases from 71.46% to 74.05%. FP decreases from 330 to 207, representing a reduction of 37.27%, whereas FN remains unchanged at 662. Therefore, the most direct evidence from this step is the reduction in false tracking outputs together with improvements in the overall association metrics. This result is consistent with using low-confidence detections to support existing trajectories, although the unchanged FN value does not independently demonstrate the recovery of additional missed targets.

Overall, the complete SeaTrack-DeepSORT configuration improves HOTA, MOTA, and IDF1 over the baseline by 1.67, 7.04, and 2.90 percentage points, respectively. FP decreases from 338 to 207, while FN decreases from 666 to 662. The progressive ablation results show that the combined configuration improves tracking performance on sequence 46, with the largest numerical change appearing after the two-stage association strategy is introduced.

To qualitatively examine the final tracking outputs, representative frames from ShipsMOT sequence 46 are presented in Figure 8. The figure compares the output of the baseline pipeline with that of SeaTrack-DeepSORT on the same video sequence. The visualization illustrates differences in the displayed trajectories and tracking boxes rather than quantifying the contribution of an individual component.

As shown in Figure 8, the baseline pipeline displays redundant or incorrect tracking outputs in frames 3 and 424, whereas fewer such outputs are visible in the corresponding SeaTrack-DeepSORT results. In frame 475, the two methods assign different numerical track labels to the displayed vessel, reflecting differences in their track association results.

To examine the behavior of SeaTrack-DeepSORT under additional maritime challenges, four ShipsMOT sequences were selected according to their dominant visual characteristics. Sequence 30 was selected for strong water-surface reflection and brightness variation, sequence 51 for foreground occlusion, sequence 63 for dense ship targets and complex background interference, and sequence 113 for low-light conditions at dusk. These sequences provide complementary scenario-based cases for qualitative and quantitative analysis. They are not treated as a statistically representative sample of the complete ShipsMOT benchmark.

As shown in Figure 9, compared with the baseline DeepSORT, SeaTrack-DeepSORT achieves significant improvements in both MOTA and HOTA across the four test sequences. Among them, the No.30 sequence obtains the most remarkable improvement, where MOTA increases from 59.87% to 98.40%, and HOTA increases from 67.36% to 83.04%. The proposed method also maintains consistent performance advantages on the remaining sequences. According to the overall results of the four sequences, SeaTrack-DeepSORT improves the average MOTA by approximately 17.32 percentage points and the average HOTA by approximately 8.99 percentage points.

Across the four selected ShipsMOT sequences, SeaTrack-DeepSORT obtains higher MOTA and HOTA than the DeepSORT baseline. These results show that the performance advantage is maintained across the evaluated sequences, although the effects of illumination, occlusion, and background interference are not measured separately.

To further illustrate the tracking behavior under the selected maritime challenge conditions, qualitative results from the same ShipsMOT sequences are presented. As shown in Figure 10, the left side presents the tracking results of the baseline DeepSORT, while the right side presents the tracking results of the proposed SeaTrack-DeepSORT. The selected sequences include typical maritime scenarios such as strong water surface reflections, target occlusions, dense multi-target distribution, and low-light conditions.

In the No.30 sequence, significant brightness variations occur in the water surface region due to strong solar reflections. The baseline DeepSORT suffers from duplicate detections and generates additional tracking trajectories, whereas SeaTrack-DeepSORT maintains stable target associations. In the No.51 sequence, the ships are partially occluded by foreground objects. The baseline DeepSORT produces duplicate trajectories under the occlusion condition, while the proposed method preserves continuous and accurate target identities. In the No.63 sequence, numerous ship targets and complex background interference are present in the scene. The baseline DeepSORT generates false trajectories, whereas SeaTrack-DeepSORT effectively suppresses incorrect track generation. In the No.113 sequence, the target visual features are significantly degraded under low-light conditions at dusk. The baseline DeepSORT suffers from missed detections, while SeaTrack-DeepSORT maintains more complete target trajectories.

The above results demonstrate that the proposed SeaTrack-DeepSORT can effectively reduce incorrect associations and trajectory losses under typical maritime challenges, including water surface reflections, target occlusions, complex backgrounds, and low illumination conditions. It achieves stronger robustness and identity preservation in complex maritime environments.

SeaTrack-DeepSORT is compared with five representative MOT algorithms, including SORT, DeepSORT, ByteTrack, StrongSORT, and BoT-SORT, on the selected ShipsMOT sequence 63. To ensure a controlled comparison, all trackers use the same SeaDet-YOLO detection results, ground-truth annotations, evaluated frame range, and TrackEval protocol. Therefore, the reported differences primarily reflect the tracking and association strategies rather than variations in detector input. The experimental results are presented in Table 6.

Under the reported evaluation protocol, SeaTrack-DeepSORT achieves HOTA, MOTA, and IDF1 values of 80.02%, 94.16%, and 97.09%, respectively. Compared with the original DeepSORT baseline, these values represent improvements of 6.40, 15.94, and 9.43 percentage points, respectively, while IDSW decreases from 5 to 0. This comparison directly supports the improvement in the complete SeaTrack-DeepSORT framework over the DeepSORT baseline under the evaluated conditions.

Compared with ByteTrack, SeaTrack-DeepSORT improves HOTA by 0.46 percentage points, MOTA by 10.69 percentage points, and IDF1 by 6.10 percentage points. Compared with BoT-SORT, the corresponding differences are 0.42, 10.80, and 6.16 percentage points. The HOTA advantages over ByteTrack and BoT-SORT are relatively small, whereas the differences in MOTA and IDF1 are more pronounced. SeaTrack-DeepSORT, ByteTrack, and BoT-SORT all obtain zero IDSW; therefore, Table 6 does not indicate a further reduction in identity switches relative to ByteTrack or BoT-SORT.

Under the reported protocol on the selected ShipsMOT sequence 63, SeaTrack-DeepSORT achieves the highest HOTA, MOTA, and IDF1 values among the compared methods while maintaining zero identity switches. These results support its effectiveness under the evaluated conditions.

Cross-dataset generalization is assessed under different maritime environments using the Singapore Maritime Dataset (SMD). SMD was constructed by Prasad et al. [34] for visual perception tasks in real-world maritime environments and is mainly used for research on maritime object detection, multi-object tracking, and intelligent maritime perception algorithms. The dataset was collected using a Canon 70D camera in the waters of Singapore and contains continuous video sequences captured under different observation platforms and environmental conditions. According to the deployment position of the camera, SMD can be divided into two categories: on-shore videos and on-board videos. The on-shore videos are collected from fixed observation platforms and are mainly used to characterize target motion patterns under stable viewing conditions. In contrast, the on-board videos are captured by cameras mounted on moving vessel platforms, reflecting viewpoint variations and image disturbances caused by platform motion during practical navigation.

The SMD dataset covers various maritime environments, including daytime, evening, foggy, and rainy conditions. It contains variations in illumination, background appearance, and target motion states, providing strong scene diversity and practical application value. In this study, both on-shore and on-board video sequences from SMD are selected for evaluation and compared with the baseline DeepSORT method, and the experimental results are presented in Table 7. By validating the proposed method on an independent dataset that is not involved in model design and training, the transfer capability, generalization performance, and robustness of SeaTrack-DeepSORT under different observation conditions and complex maritime environments are further evaluated.

SeaTrack-DeepSORT achieves superior tracking performance compared with DeepSORT under both observation conditions. In the on-shore videos, the target motion is relatively stable due to the fixed viewpoint, allowing DeepSORT to accomplish basic target tracking. However, association errors still occur during the tracking process. SeaTrack-DeepSORT improves MOTA from 40.89% to 46.06% and HOTA from 46.36% to 48.08%, while reducing IDSW from 15 to 1. The reduction in IDSW from 15 to 1 indicates fewer identity changes on the selected on-shore sequences. In the on-board videos, the tracking task becomes more challenging because camera motion and target position displacement are introduced by the movement of the imaging platform. Compared with DeepSORT, SeaTrack-DeepSORT improves MOTA from 48.50% to 58.93%, representing an increase of 10.43 percentage points. Meanwhile, HOTA increases from 60.13% to 68.38%, improving by 8.25 percentage points, and IDSW is reduced from 1 to 0. For the selected on-board sequences, these metric changes show that the complete framework performs better than DeepSORT under moving-camera conditions.

Considering both test scenarios, the average MOTA of SeaTrack-DeepSORT increases from 44.70% to 52.50%, representing an improvement of 7.80 percentage points. The average HOTA increases from 53.25% to 58.23%, with an improvement of 4.98 percentage points, while the average IDSW decreases from 8 to 0.5. These results provide preliminary cross-dataset evidence that SeaTrack-DeepSORT retains an advantage over DeepSORT on the selected SMD on-shore and on-board sequences.

## 5. Conclusions

This study developed SeaTrack-DeepSORT to address observation variability and platform-induced displacement in maritime video tracking. The framework combines a modified YOLOv11 detector with a DeepSORT-based tracking pipeline. Its design connects multiscale observation generation, confidence-dependent state updates, correction of predicted target centers, and low-confidence track recovery. The work contributes a specific adaptation and integration of these operations for ship detection and tracking under the evaluated maritime conditions.

First, SeaDet-YOLO is constructed by adapting the YOLOv11 architecture with the ADown operation and an additional P2 detection branch. To address the challenges of small-scale targets, complex background interference, and target scale variations, SeaDet-YOLO enhances multi-scale feature representation through structural improvements. Meanwhile, the model maintains lightweight characteristics while achieving higher detection accuracy. Experimental results demonstrate that SeaDet-YOLO outperforms several mainstream detection algorithms on the WSODD dataset, verifying its effectiveness for ship detection in complex water surface environments.

Second, to overcome the limitations of conventional DeepSORT under maritime conditions, including uncertain target motion, camera motion from vessel platforms, and short-term detection failures, SeaTrack-DeepSORT is built upon the original DeepSORT framework and integrates confidence-dependent Kalman updating, ECC-Affine camera-motion compensation, and two-stage association. Experimental results show that SeaTrack-DeepSORT significantly improves key tracking metrics, including HOTA, MOTA, and IDF1, while reducing identity switches compared with the original DeepSORT. It achieves better trajectory continuity and identity preservation.

Furthermore, experiments on multiple selected ShipsMOT sequences and selected SMD on-shore and on-board sequences show that SeaTrack-DeepSORT retains an advantage over DeepSORT under the evaluated maritime conditions. The qualitative results also illustrate fewer redundant and incorrect tracking outputs in the selected frames. These findings provide initial evidence of cross-scene and cross-dataset transfer, while broader validation on complete benchmarks remains necessary.

In conclusion, the integrated SeaDet-YOLO and SeaTrack-DeepSORT framework improves ship detection and multi-object tracking under the evaluated maritime conditions. The framework is relevant to applications such as autonomous perception of unmanned surface vehicles, maritime target surveillance, and intelligent water management. Future research will focus on further lightweight optimization, embedded deployment, and validation on real unmanned vessel platforms to improve real-time performance and engineering applicability under resource-constrained conditions.

## Figures and Tables

**Figure 1:**
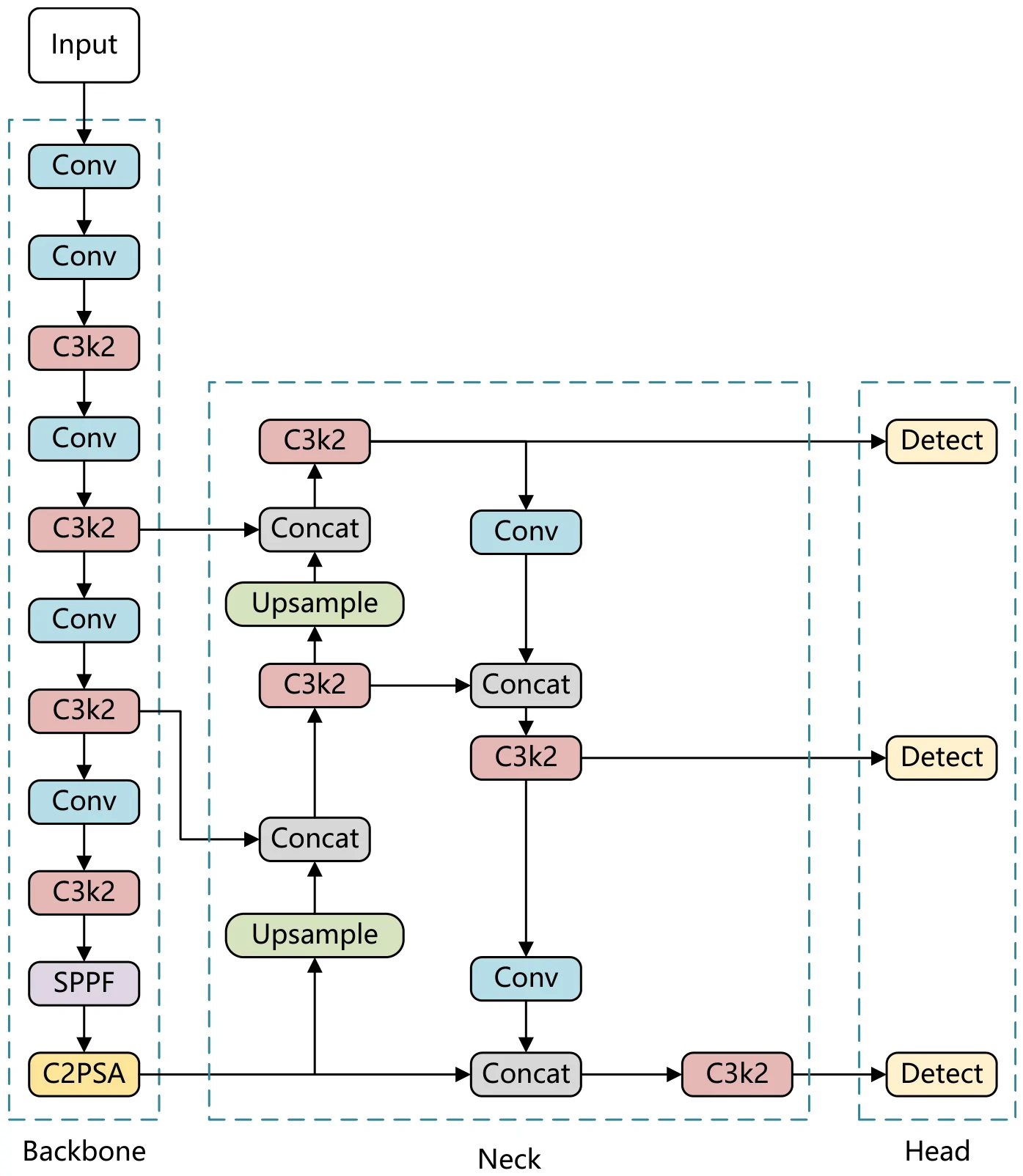
YOLOv11 Network Structure.

**Figure 2:**
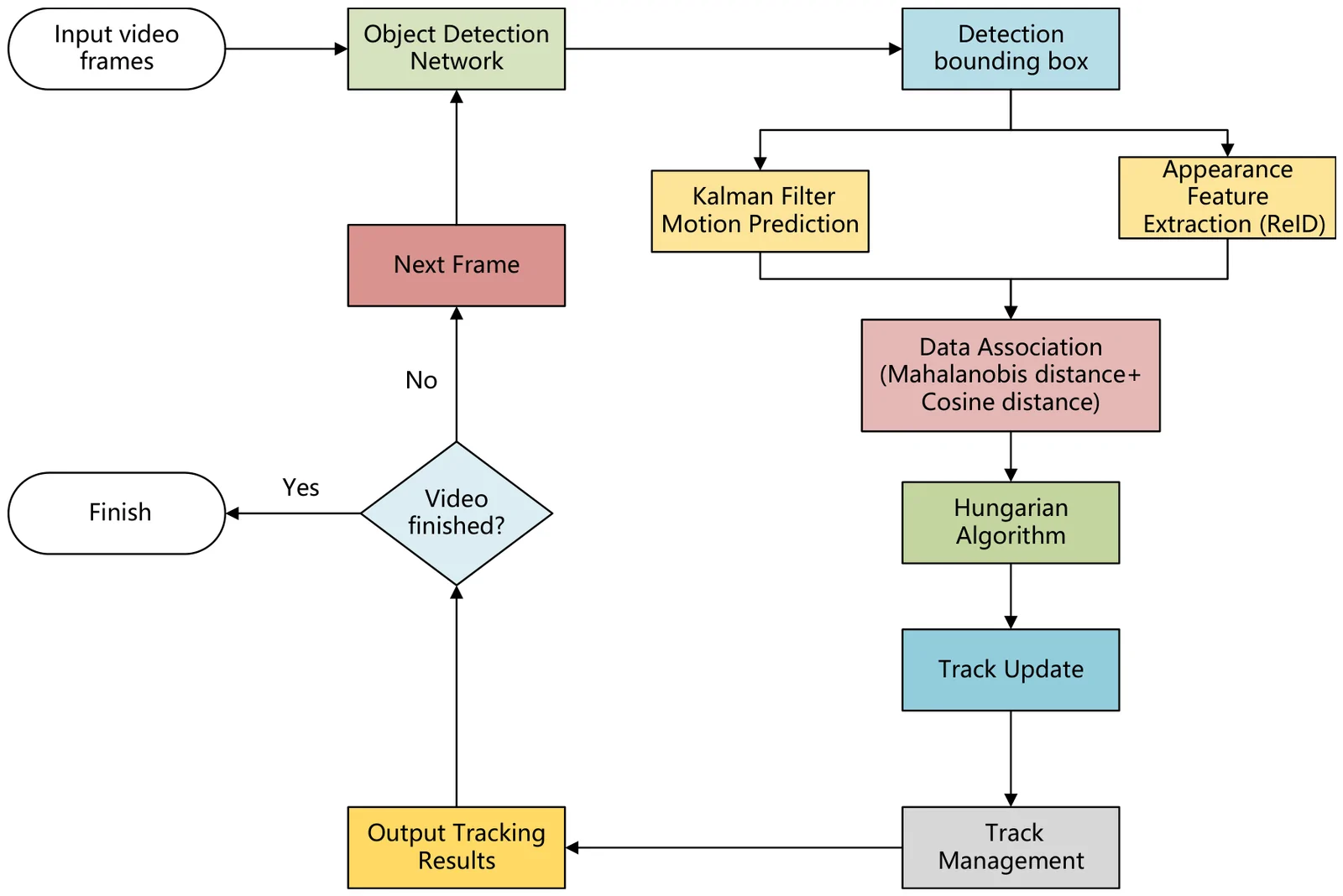
DeepSORT flowchart.

**Figure 3:**
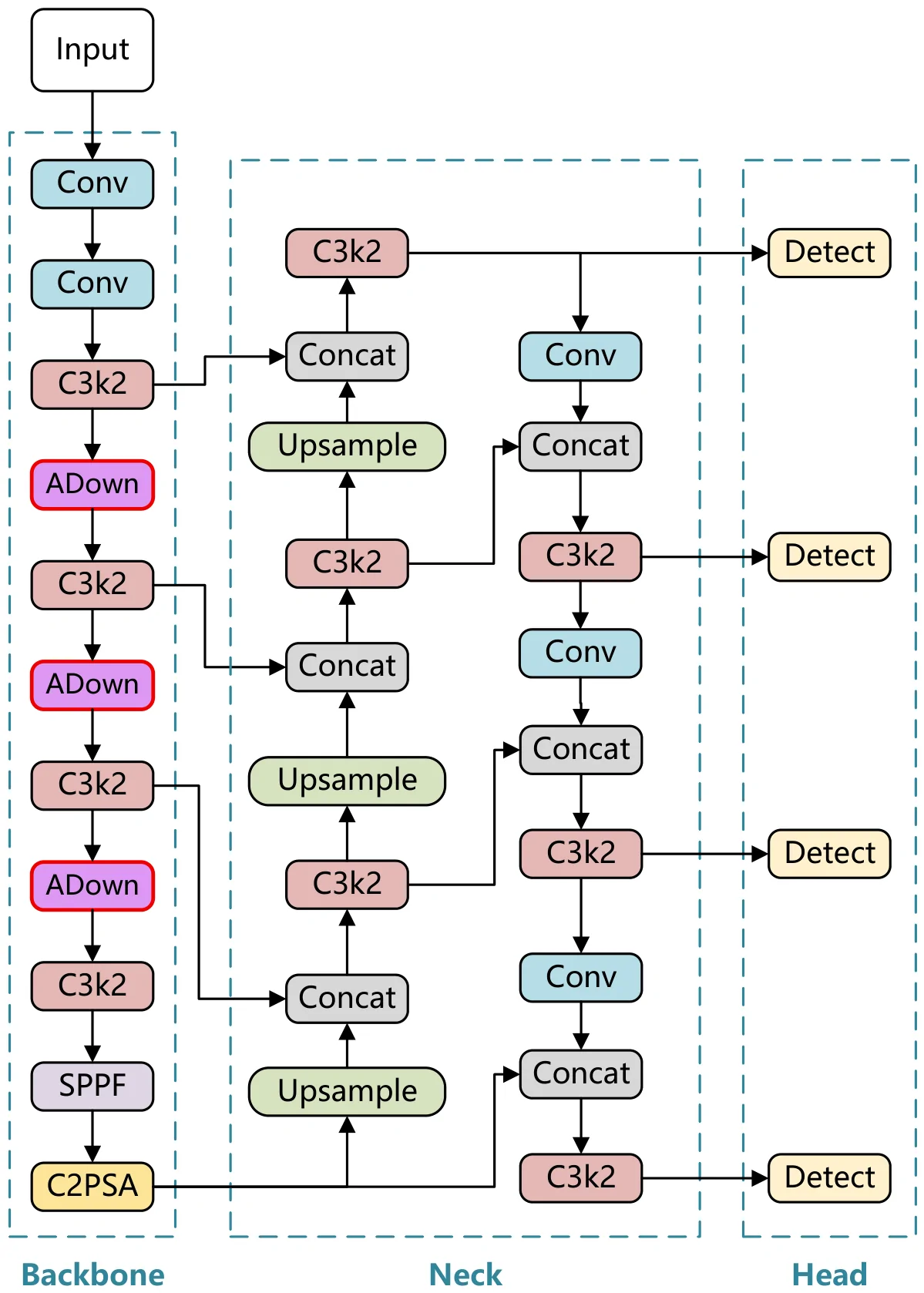
SeaDet-YOLO Network Structure.

**Figure 4:**
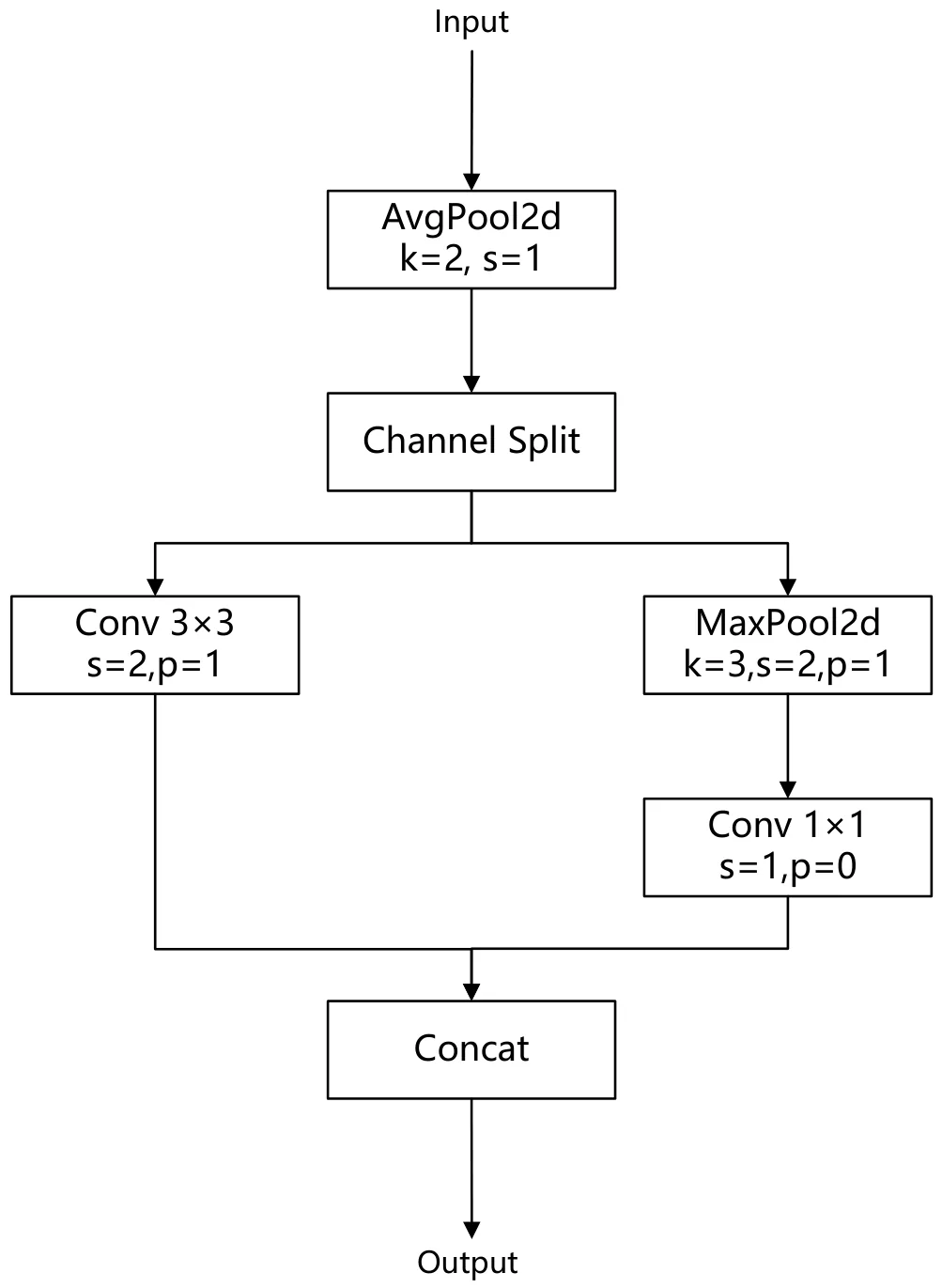
Architecture of the ADown downsampling module.

**Figure 5:**
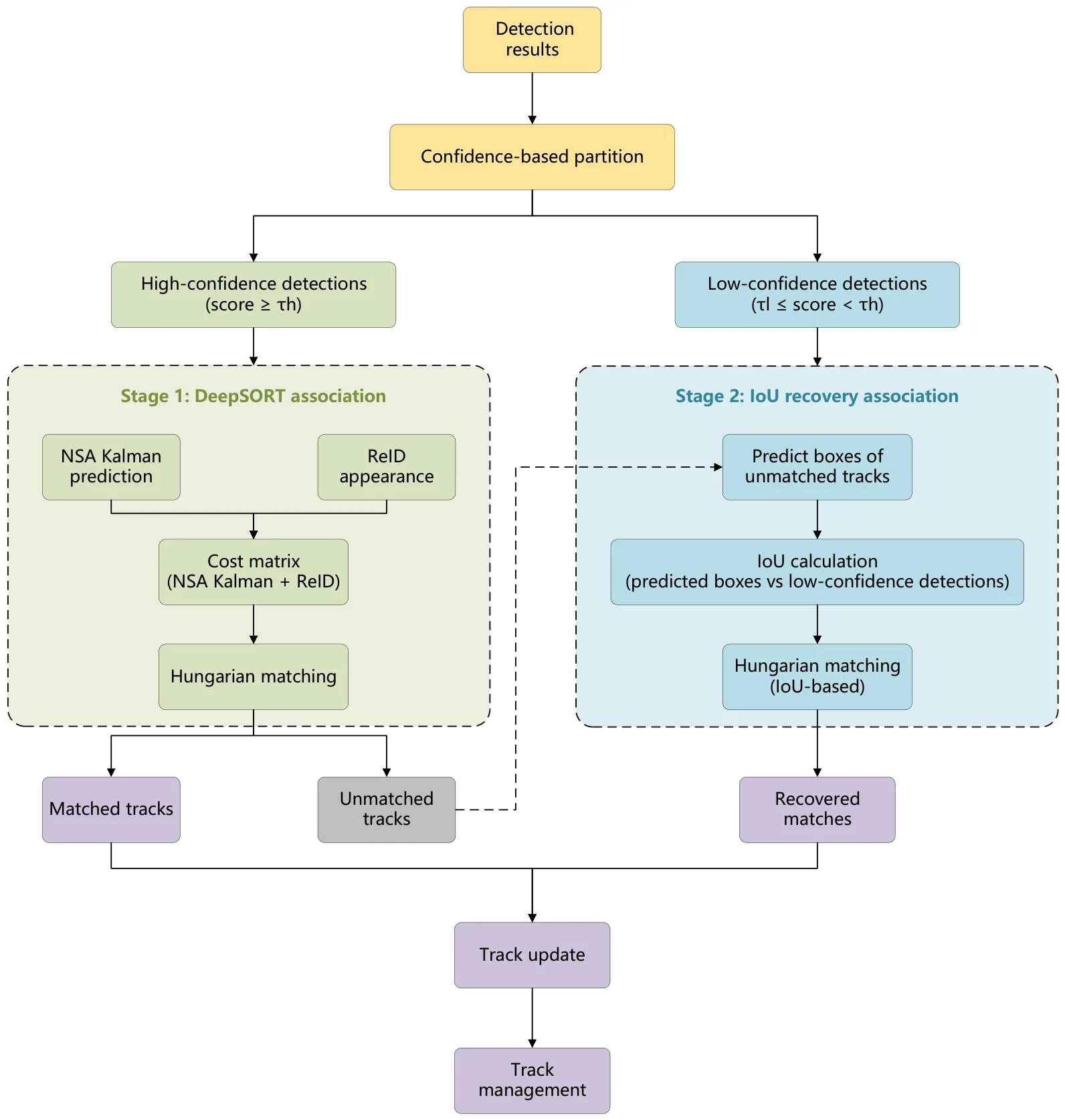
Flowchart of the two-stage association strategy.

**Figure 6:**
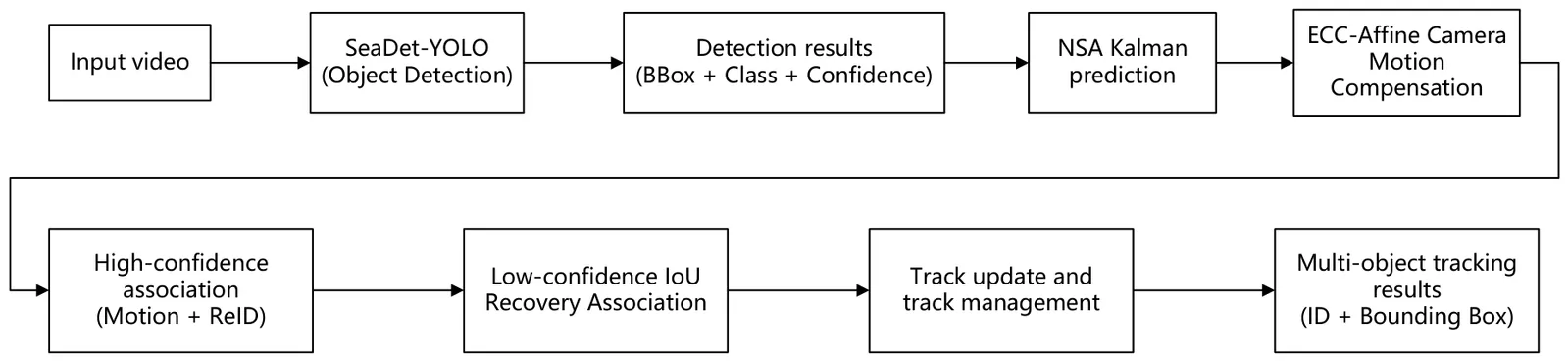
Integrated SeaDet-YOLO and SeaTrack-DeepSORT framework.

**Figure 7:**
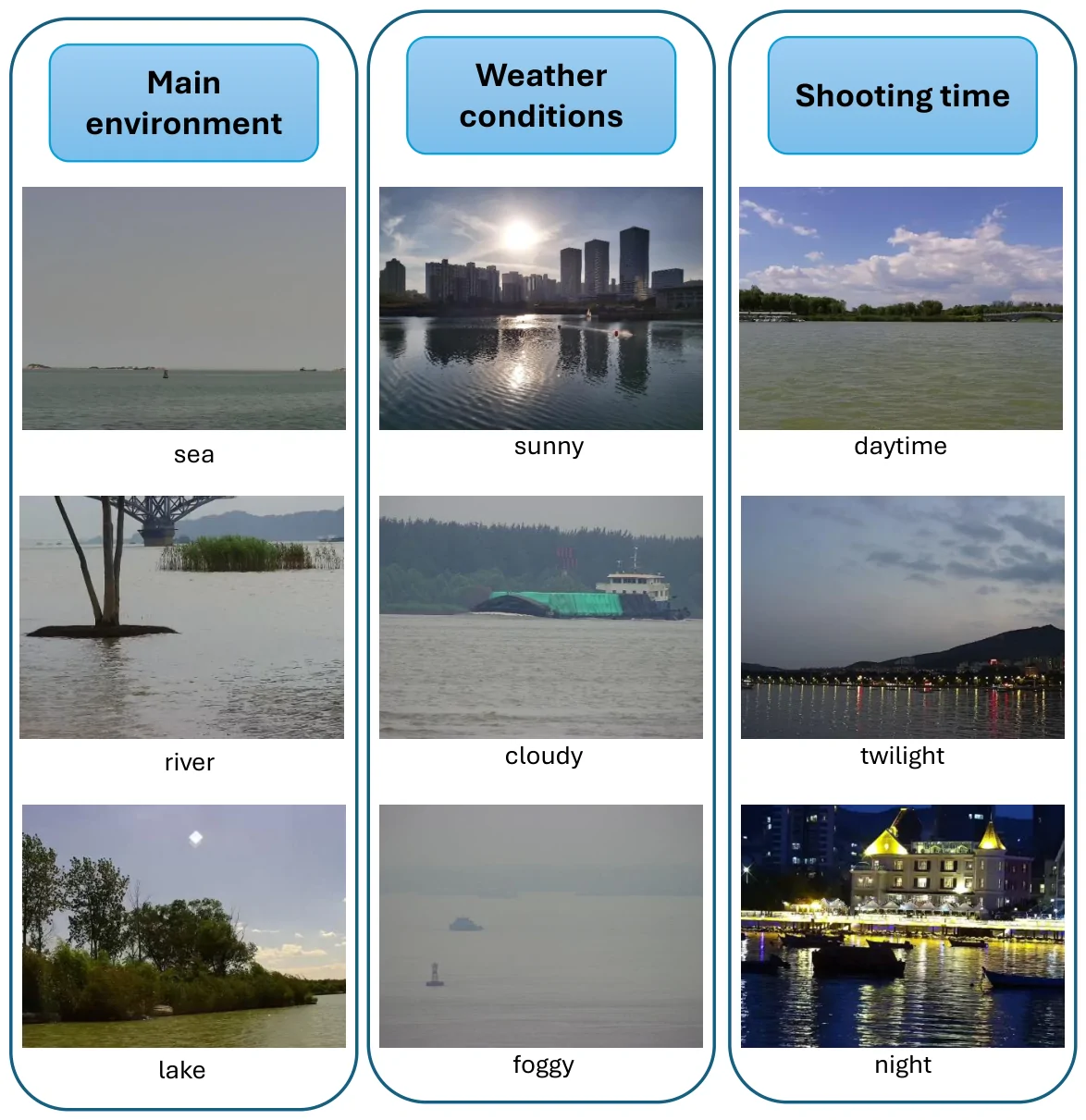
Typical environments and conditions in WSODD.

**Figure 8:**
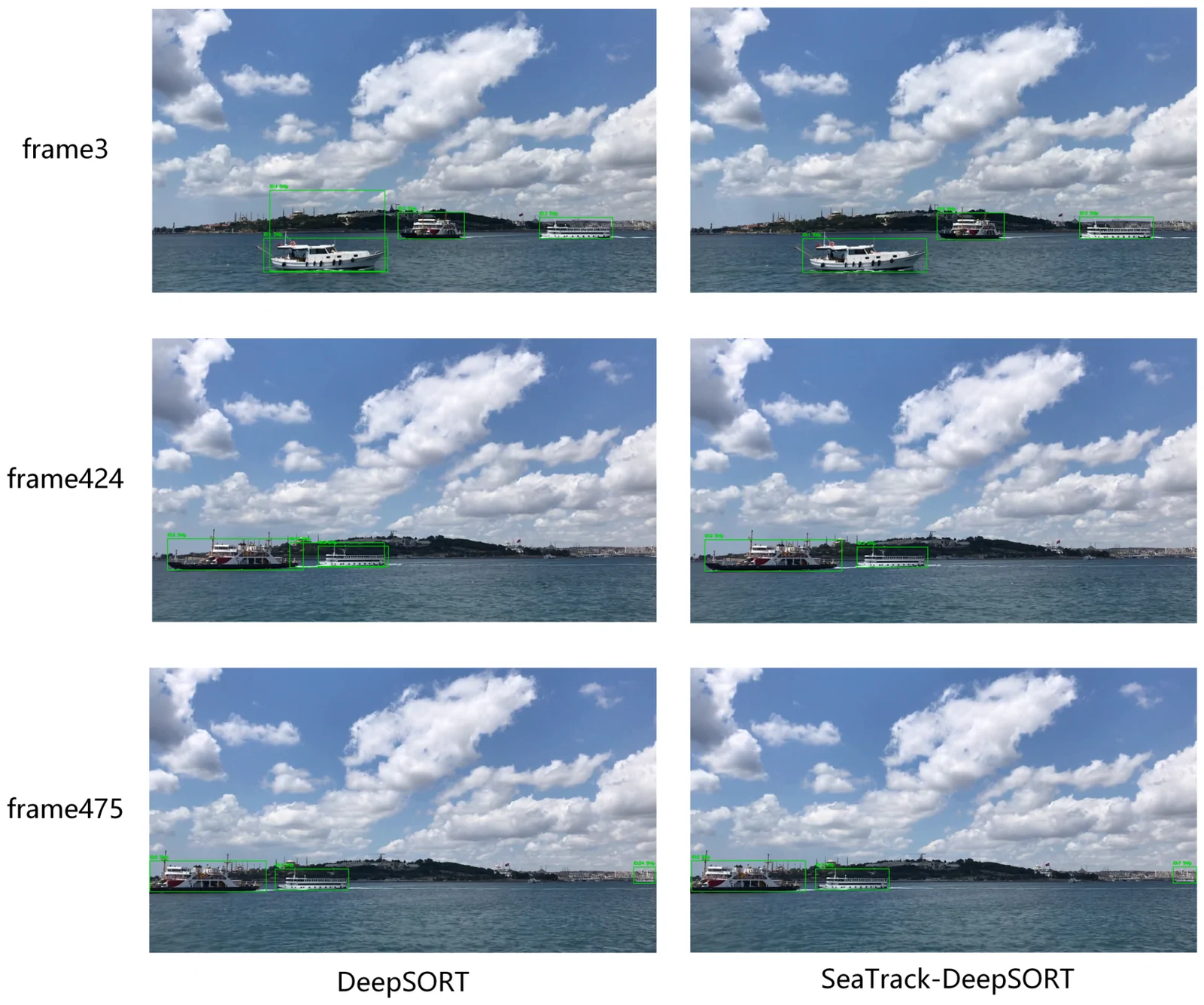
Qualitative comparison of tracking results between DeepSORT and SeaTrack-DeepSORT on sequence 46.

**Figure 9:**
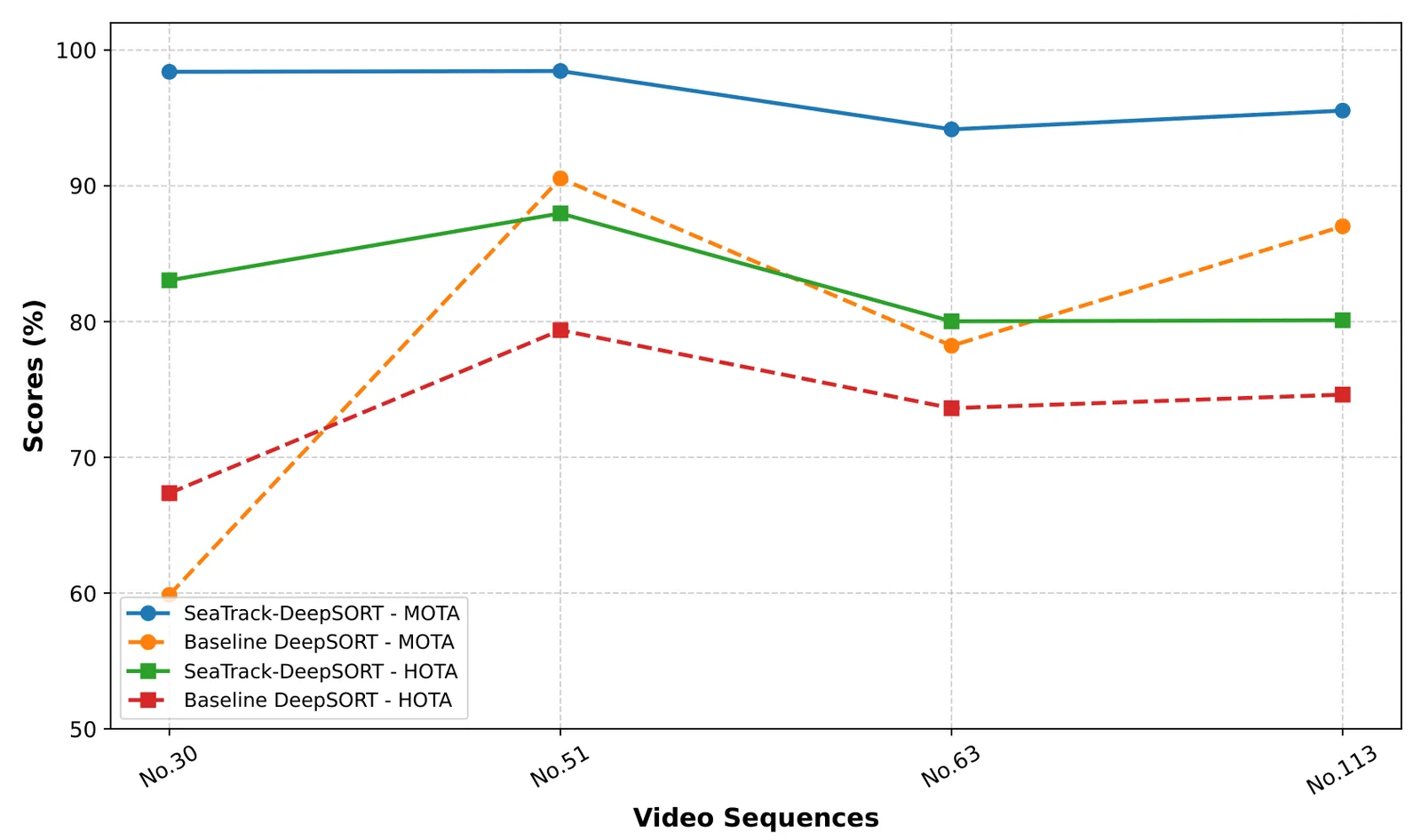
Multi-sequence tracking comparison.

**Figure 10:**
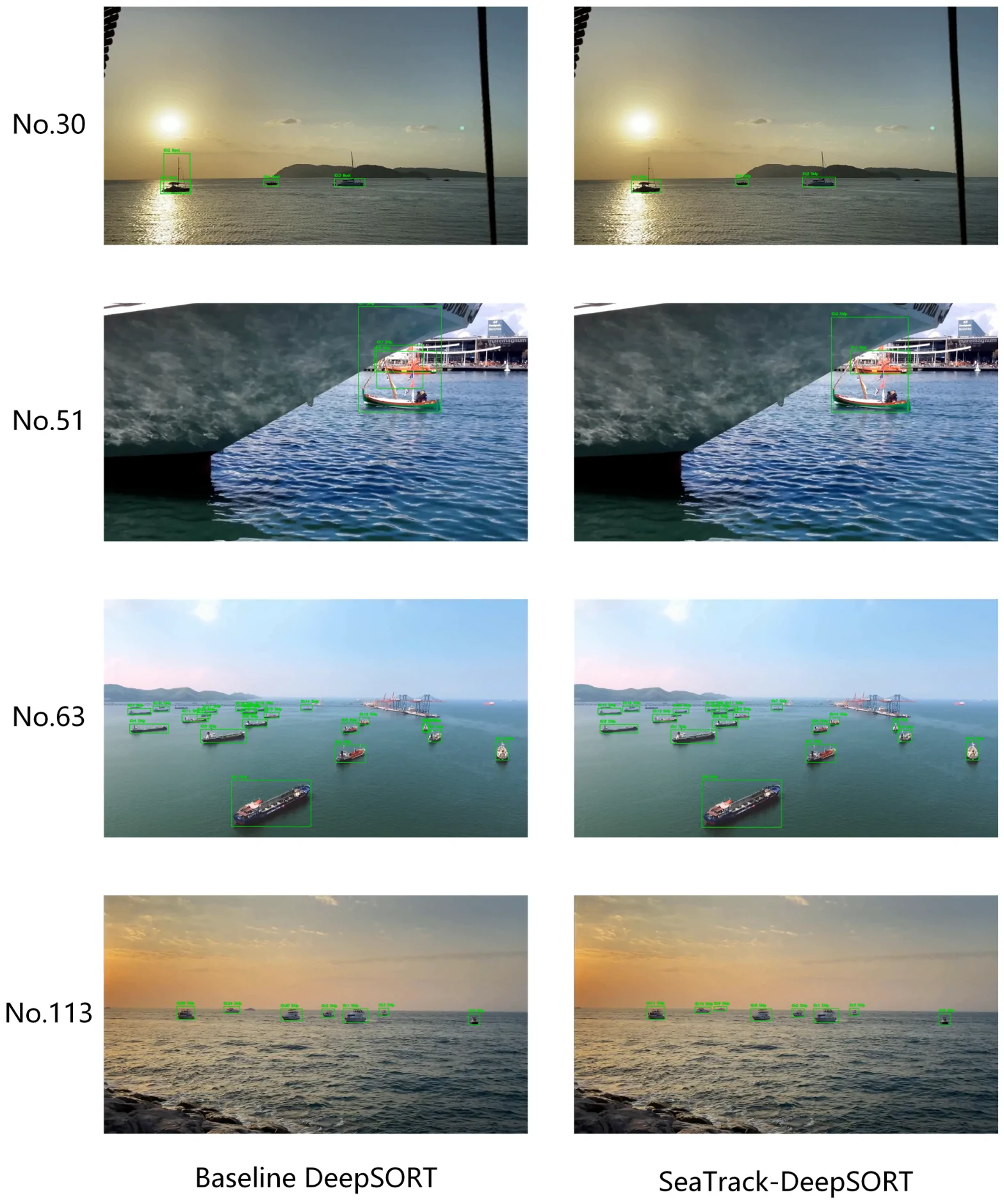
Visualization of tracking results on representative sequences of the ShipsMOT dataset.

**Table 1:** Principal parameter settings of SeaTrack-DeepSORT.

Parameter	Value
Confidence-scaling coefficient α	0.3
High-confidence threshold $\tau_{\mathrm{high}}$	0.5
Low-confidence threshold $\tau_{\mathrm{low}}$	0.1
Maximum cosine distance	0.2
Mahalanobis gating threshold	9.4877
Maximum IoU distance	0.7
Track confirmation parameter $n_{\mathrm{init}}$	3
Maximum track age	70
Appearance-feature budget	100

**Table 2:** Evaluation results of SeaDet-YOLO compared with different methods.

Model	P (%)	R (%)	F1 (%)	mAP_50_ (%)	mAP_50:95_ (%)	Params (M)	GFLOPs
SSD	59.7	57.1	58.4	61.7	27.6	26.3	31.1
Faster R-CNN	58.5	55.4	56.9	60.6	25.4	41.1	139.8
YOLOv5n	74.8	61.5	67.7	67.9	36.7	2.2	5.9
YOLOv6n	68.1	63.9	66.0	66.8	32.6	4.1	11.6
YOLOv8n	76.5	65.5	70.6	70.1	39.1	2.7	7.0
YOLOv10n	65.4	60.2	62.7	66.4	35.1	2.7	8.4
YOLOv11n	80.55	69.97	74.89	76.51	43.07	2.6	6.5
SeaDet-YOLO	84.17	73.43	78.43	80.27	46.02	2.3	9.6

**Table 3:** Ablation study results of different components in SeaDet-YOLO.

Model	P (%)	R (%)	F1 (%)	mAP_50_ (%)	mAP_50:95_ (%)	Params (M)	GFLOPs
YOLOv11n	80.55	69.97	74.89	76.51	43.07	2.6	6.5
YOLOv11n + ADown	81.54	70.78	75.78	76.94	44.03	2.2	5.6
YOLOv11n + P2	80.71	72.36	76.32	78.90	44.53	2.6	6.4
SeaDet-YOLO	84.17	73.43	78.43	80.27	46.02	2.3	9.6

**Table 4:** Tracking performance comparison between YOLOv11 and SeaDet-YOLO on the ShipsMOT dataset.

Algorithm	Video Sequence	MOTA (%)	HOTA (%)	IDSW
YOLOv11 + DeepSORT	46	34.40	41.34	13
	73	96.24	87.90	13
	Average	65.32	64.62	13
SeaDet-YOLO + DeepSORT	46	47.27	53.33	0
	73	98.23	90.40	5
	Average	72.75	71.87	2.5

**Table 5:** Progressive configuration comparison of SeaTrack-DeepSORT on ShipsMOT.

Configuration	NSA	ECC-CMC	TSA	HOTA (%)	MOTA (%)	IDF1 (%)	FP	FN
DeepSORT	×	×	×	53.33	47.27	71.15	338	666
NSA Kalman	✓	×	×	53.46	47.37	71.17	335	666
NSA Kalman + ECC	✓	✓	×	53.47	47.90	71.46	330	662
SeaTrack-DeepSORT	✓	✓	✓	55.00	54.31	74.05	207	662

Note: ✓ indicates that the corresponding component is included in the configuration, whereas × indicates that it is not included.

**Table 6:** Comparison of different multi-object tracking algorithms on the ShipsMOT dataset.

Method	HOTA (%)	MOTA (%)	IDF1 (%)	IDSW
SORT	79.42	83.81	90.46	6
DeepSORT	73.62	78.22	87.66	5
ByteTrack	79.56	83.47	90.99	0
StrongSORT	74.00	77.31	85.38	29
BoT-SORT	79.60	83.36	90.93	0
SeaTrack-DeepSORT	80.02	94.16	97.09	0

**Table 7:** Cross-dataset comparison on the selected on-shore and on-board sequences of SMD.

Algorithm	Video	MOTA (%)	HOTA (%)	IDSW
DeepSORT	On-shore	40.89	46.36	15
	On-board	48.50	60.13	1
	Average	44.70	53.25	8
SeaTrack-DeepSORT	On-shore	46.06	48.08	1
	On-board	58.93	68.38	0
	Average	52.50	58.23	0.5

## Data Availability

The datasets used in this study are publicly available from their original sources.
